# Blood eosinophil count is associated with early atherosclerotic artery changes in asthma

**DOI:** 10.1186/s12890-024-03322-0

**Published:** 2024-10-11

**Authors:** Leonie Biener, Ben Christoph Frisch, Dirk Skowasch, Carmen Pizarro, Andrea Budimovska, Georg Nickenig, Max Jonathan Stumpf, Nadjib Schahab, Christian Schaefer

**Affiliations:** Department of Internal Medicine II, Cardiology, Pneumology and Angiology, University Hospital Bonn, Bonn, Germany

**Keywords:** Asthma, Atherosclerosis, Eosinophilia, Eosinophils, Strain analysis

## Abstract

**Objective:**

Asthma is linked to atherosclerosis, yet the underlying mediators remain elusive. Eosinophils may contribute to both asthmatic and atherosclerotic inflammation. Hence, this study aimed to explore the potential associations of eosinophils with artery changes among patients with asthma.

**Methods:**

We assessed strain values of the common carotid arteries (CCAs) via vascular speckle tracking and compared asthma patients with low (< 300/µl) and high (≥ 300/µl) blood eosinophil counts (BEC).

**Results:**

We enrolled 100 patients, 42 with a BEC of < 300 and 58 with a BEC of ≥ 300 n/µl. Patients with high BEC exhibited more severe disease, characterized, e.g., by a higher frequency of acute exacerbations (1.3 ± 2.1 vs. 2.6 ± 2.4 n/year, *p* = 0.005). Both groups presented similar profiles in terms of conventional cardiovascular risk. The high BEC group demonstrated elevated arterial stiffness, reflected by reduced radial strain (mean radial strain of the right CCA: 2.7 ± 1.4% for BEC ≥ 300 n/µl vs. 3.5 ± 1.7% for BEC < 300 n/µl, *p* = 0.008; left CCA: 2.6 ± 1.4% vs. 4.1 ± 2.2%, *p* < 0.001). A weak yet statistically significant negative correlation was observed between BEC and radial strain for the right CCA (R2 = 0.131, b=-0.001, *p* = 0.001) and left CCA (R2 = 0.086, b=-0.001, *p* = 0.015). However, the prevalence of cerebrovascular disease was similar in both groups (31,0% vs. 50,0%, *p* = 0.057).

**Conclusion:**

We identified a correlation between BEC and vascular stiffness, which supports the hypothesis that eosinophils may promote atherosclerosis.

**Clinical trial number:**

Due to the exploratory and predominantly retrospective nature of the study, trial registration was not conducted. The only prospective procedure conducted was the angiological sonography to evaluate the current state. No ensuing health-related interventions were performed specifically for this study.

## Introduction

Asthma is an obstructive airway disease characterized by inflammation of the bronchial airways and affects approximately 10% of adults and 5% of children worldwide [1]. The chronic systemic inflammation observed in conditions such as asthma has been linked to cardiovascular diseases and events [2–5]. Despite this recognized association, the precise pathomechanisms driving atherosclerosis development in individuals with asthma remain poorly understood [6, 7]. While atherosclerosis has traditionally been associated with type 1 inflammation, recent studies have underscored the contribution of type 2 inflammation to its pathogenesis. Eosinophil granulocytes, known as key players in type 2 inflammation associated with asthma, have been implicated in promoting atherosclerosis [8, 9]. Therefore, our study aimed to explore the relationship between eosinophils and atherosclerosis in asthma patients. Specifically, we aimed to assess early atherosclerotic vessel changes via strain analysis based on the blood eosinophil count in patients with asthma.

## Methods

### Patient characteristics

The study adhered to the principles outlined in the Declaration of Helsinki and received approval from the local ethics committee at the Medical Faculty of the University of Bonn (No 117/22). All participants provided written informed consent. This study was conducted between July 2022 and April 2023 and involved 100 consecutive asthma patients, aged 18 years and above, attending the Pneumological Outpatient Department of the University of Bonn, with a physician diagnosed asthma. The exclusion criteria included lack of consent or inability to provide consent, along with comorbidities involving hypereosinophilia, namely hypereosinophilic syndrome and eosinophilic granulomatosis with polyangiitis (EGPA). During the examination, all patients presented with stable disease without exacerbation.

**Fig. 1:**
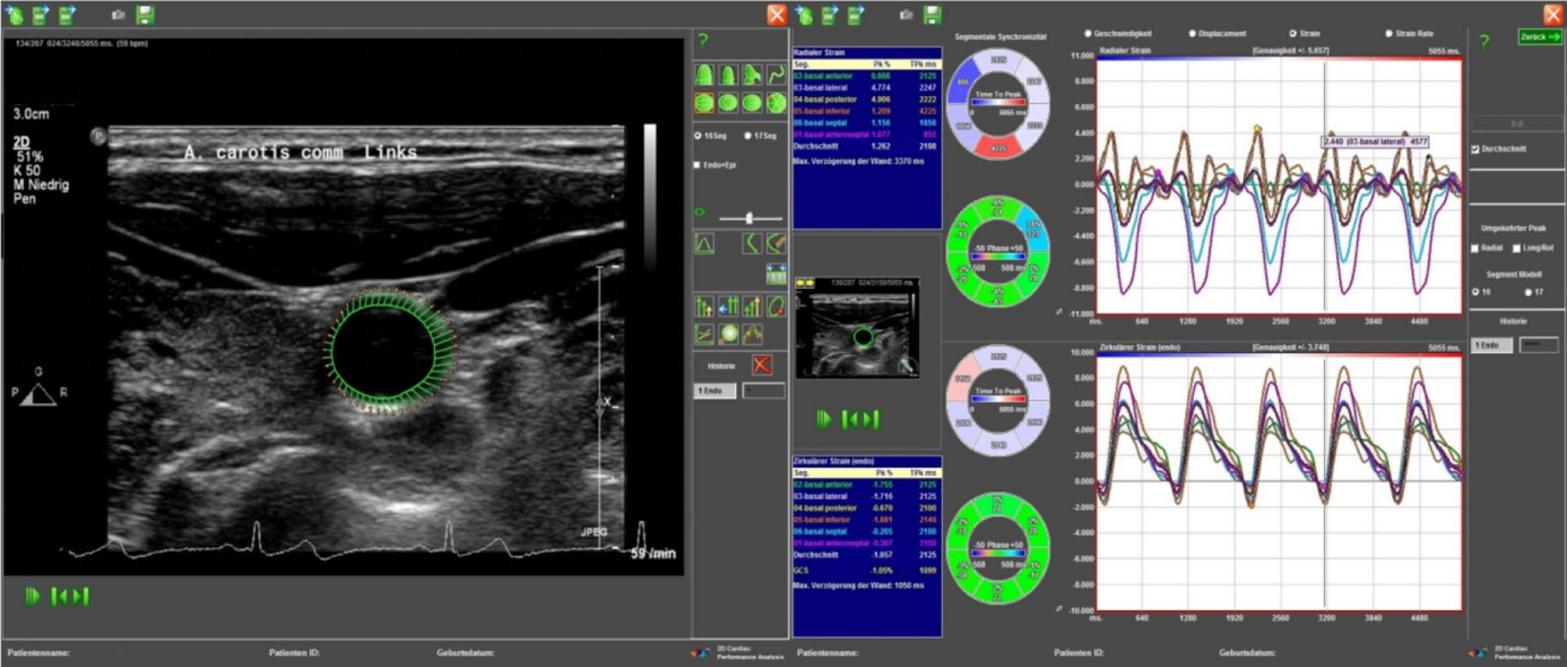
Strain analysis (radial strain) was carried out with ImageArena Version 4.6, TomTec Systems. The green area around the vessel on the left represents the radial expansion of the left common carotid artery

Additional patient characteristics, including medical history, medication, laboratory parameters, and lung function values, were retrieved from the patients’ medical records in routine clinical practice. An exacerbation was defined as a (sub-)acute worsening of the patient’s symptoms and/or lung function that necessitated OCS burst therapy [1].

The subjects were categorized into two groups on the basis of blood eosinophil count (BEC) as recommended by the German guidelines (≥ 300/µl vs. < 300/µl) [10]. Blood eosinophil counts were obtained outside of an exacerbation and without the influence of systemic corticosteroid therapy or biologic therapy [10].

**Fig. 2:**
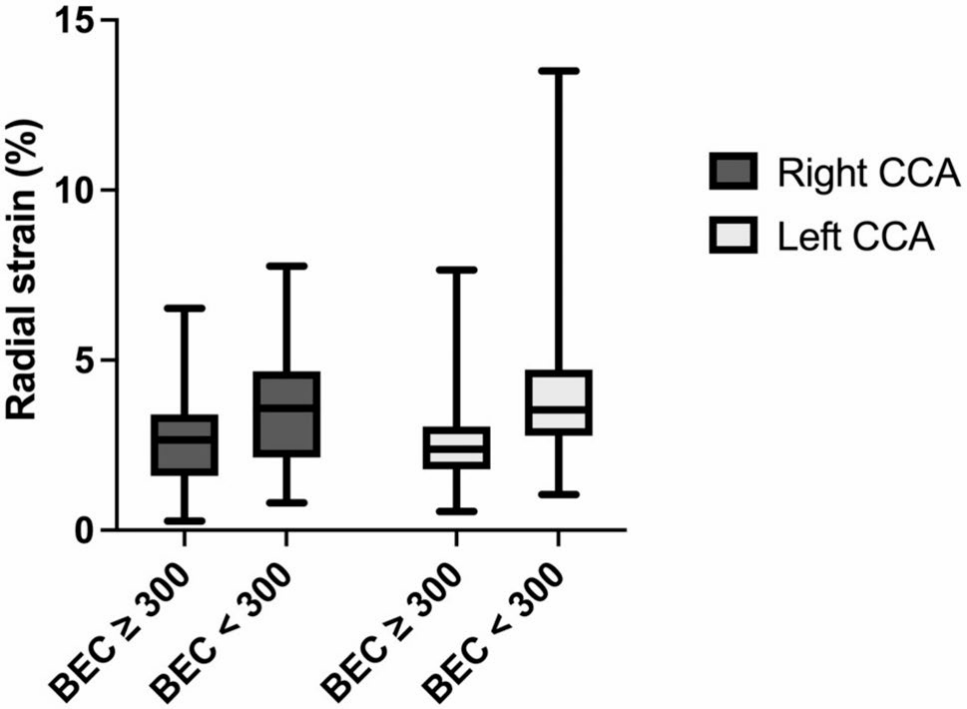
The mean radial strain (%) of the left common carotid artery (CCA) and the right CCA was compared between asthma patients with a blood eosinophil count (BEC) of ≥ 300 and those with a blood eosinophil count of < 300 n/µl. The radial strain values for the BEC ≥ 300 group were significantly lower (2.7 ± 1.4 vs. 3.5 ± 1.7, *p* = 0.008 for the right CCA; 2.6 ± 1.4 vs. 4.1 ± 2.2, *p* < 0.001 for the left CCA)

**Fig. 3:**
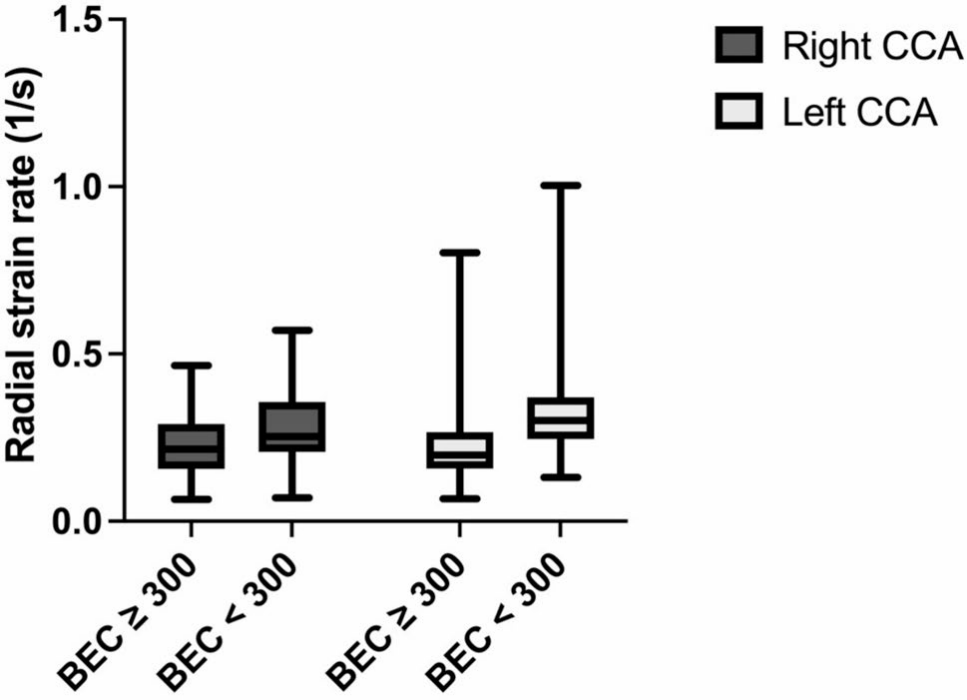
The mean radial strain rates (1/s) of the left common carotid artery (CCA) and the right CCA were compared between asthma patients with blood eosinophil counts (BECs) ≥ 300 and those with BECs < 300 n/µl. The radial strain rates for the BEC ≥ 300 group were significantly lower (0.23 ± 0.09 vs. 0.28 ± 0.10, *p* = 0.006 for the right CCA; 0.23 ± 0.13 vs. 0.32 ± 0.15, *p* = 0.006 for the left CCA)

### Vascular assessments

All patients underwent ultrasonographic examination of both the right and left common carotid arteries (CCAs) via the Philips iE33 ultrasonic device.

**Table 1:** Patient characteristics

Variable	Eos < 300 *n*/µl (*n* = 42)	Eos ≥ 300 *n*/µl (*n* = 58)	*p*-value
Age (years)	54.7 ± 16.2	57.6 ± 14.2	0.354^a^
Female	32 (76.2%)	30 (51.7%)	0.013* ^**c**^
BMI (kg/m²)	26.4 ± 4.6	28.4 ± 7.1	0.089^b^
Ever-smoker	17 (40.5%)	27 (53.4%)	0.546^c^
Packyears	9.1 ± 16.4	9.4 ± 15.1	0.924^a^
Hypercholesterolemia	13 (31.0%)	18 (31.0%)	0.993^c^
Arterial hypertension	17 (40.5%)	27 (46.6%)	0.546^c^
Diabetes	3 (7.1%)	8 (13.8%)	0.350^d^
Family history for CV diseases	16 (38.1%)	16 (27.6%)	0.266^c^
OSAS	1 (2.4%)	6 (10.3%)	0.234^d^
Years since asthma diagnosis	24.9 ± 17.8	22.7 ± 17.0	0.533^a^
ACT-Score	15.9 ± 5.5	13.3 ± 5.9	0.038* ^**a**^
Exacerbations (n/year)	1.3 ± 2.1	2.6 ± 2.4	0.005* ^**a**^
FeNO (ppB)	33.1 ± 44.3	47.3 ± 41.3	0.162^a^
IgE (U/ml)	404.8 ± 651.8	696.0 ± 1623.0	0.297^a^
FeV1 (% predicted)	77.7 ± 19.6	65.5 ± 18.9	0.002* ^**a**^
Severe Asthma^+^	20 (47.6%)	50 (86.2%)	< 0.001* ^**c**^
ICS	39 (92.9%)	56 (96.6%)	0.518^d^
- Low-dose	10 (23.8%)	9 (15.5%)	
- Medium-dose	14 (33.3%)	20 (34.5%)	
- High-dose	15 (35.7%)	27 (46.6%)	
LABA	37 (88.1%)	55 (94.8%)	0.275^d^
LAMA	22 (52.4%)	41 (70.7%)	0.061^c^
Maintenance OCS therapy	7 (16.7%)	9 (15.5%)	0.877^c^
- OCS dose (mg/d prednisolone equivalent)	5 [4;7]	5 [3;12.5]	0.758^e^
IL5-R-therapy	1 (2.4%)	29 (50.0%)	< 0.001* ^**c**^

Data shown as n (%), medium value ± standard deviation or if not normally distributed as median [25%-percentile;75%-percentile]

* = *p* < 0.05 = statistically significant

^a^ t-test for independent variables. ^b^ Welch-test. ^c^ chi-square test. ^d^ Fisher’s exact test

^e^ Mann-Whitney-U-test

^+^ defined by the need for high-dose inhaled corticosteroid (ICS) therapy and another medication to be controlled, or asthma that remains uncontrolled despite this therapy, according to the ERS/ATS guidelines [36]

ACT = asthma control test. BMI = body mass index. CV = cardiovascular. FeNO = fraction of exhaled nitric oxide. FEV1 = forced expiratory volume. ICS = inhaled corticosteroids. IgE = Immunoglobuline E in serum. IL-5-(R) = interleukine 5 (receptor). LABA = long-acting beta-2-agonist. OCS = oral corticosteroid. OSAS = obstructive sleep apnea. LAMA = long-acting muscarinic antagonist

The vessels were examined for the presence of vascular plaques, which were the defining characteristic of cardiovascular disease.

**Fig. 4:**
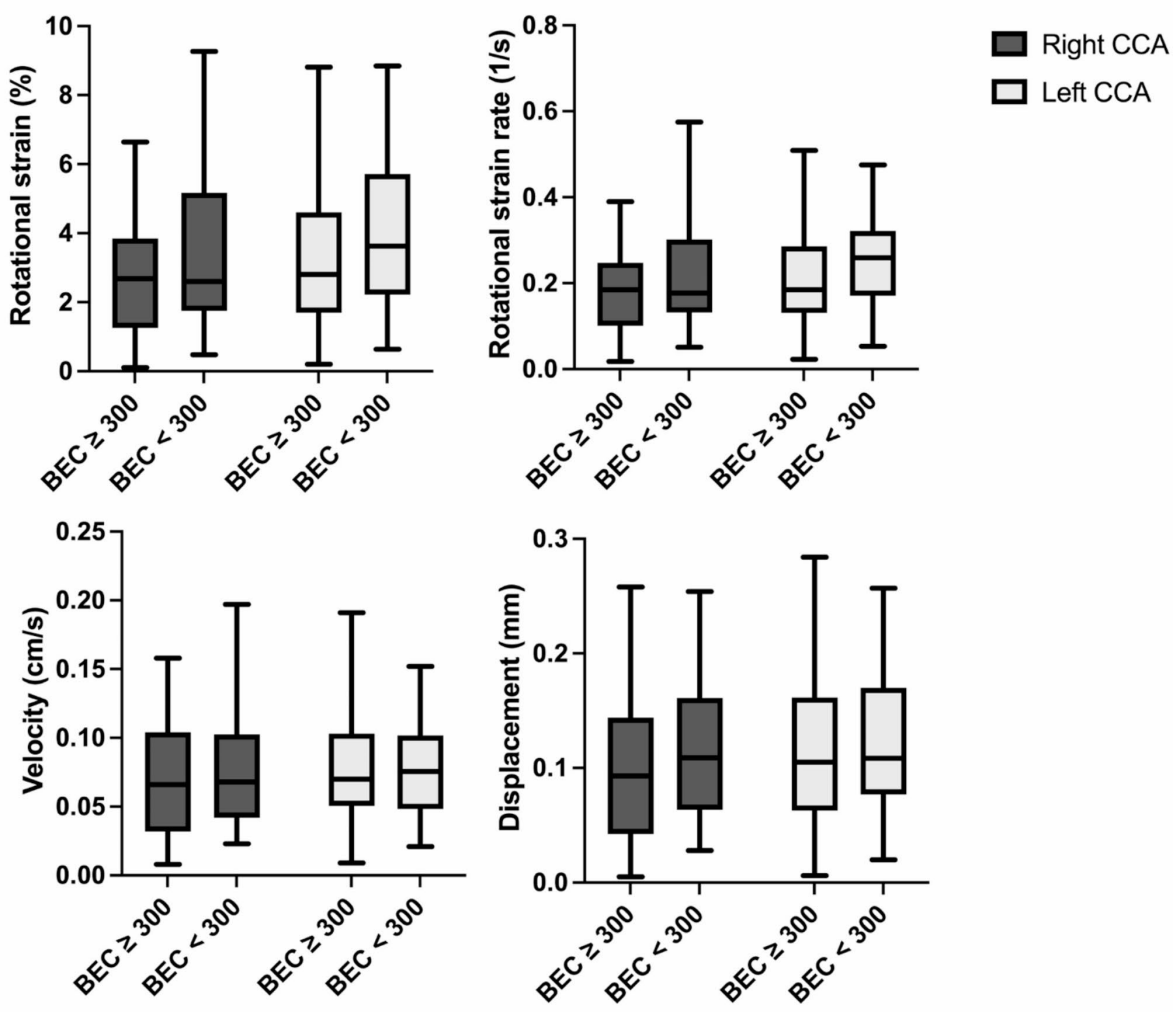
The rotational strain, rotational strain rate, velocity, and displacement of the common carotid artery (CCA) did not significantly differ between asthma patients with a blood eosinophil count (BEC) of ≥ 300 and those with a BEC of < 300 n/µl. Rotational strain (right CCA *p* = 0.099, Welch test. Left CCA *p* = 0.099), rotational strain rate (right CCA *p* = 0.085, Welch test. Left CCA *p* = 0.186), velocity (right CCA *p* = 0.330, left CCA *p* = 0.850), and displacement (right CCA *p* = 0.111, left CCA *p* = 0.469)

Furthermore, the arteries were examined in the short axis, and an electrocardiogram (ECG)-triggered recording was made over at least five cardiac cycles with the patient holding their breath and with slight compression of the internal jugular vein. The recordings were subsequently analyzed via ImageArena^®^ Version 4.6 software by TomTec Systems GmbH, Munich, Germany, following previously described methods [11]. The analysis included the radial strain (radial expansion of the vessel during one cardiac cycle in %), circumferential strain (change in the vessel wall circumference during one heart cycle in %), radial strain rate and circumferential strain rate (dynamic parameters of vessel wall motion over time (1/s)), radial displacement (overall movement of the vessel wall in millimeters (mm)) and radial velocity in centimeters per second (cm/s)).

The primary outcome measure was the radial strain, as illustrated in Fig. 1, which is an example of a strain analysis.

All measurements were performed by experienced examiners, who were blinded to the results of the blood eosinophil count.

### Statistical analysis

The data were analyzed via IBM^®^ SPSS^®^ Statistics, version 29.0. Graphs were created with GraphPad Prism 10.1.1, MacKiev^®^ 1994–2023 GraphPad Software, LLC. Continuous variables are presented as the means ± standard deviations, whereas categorical variables are expressed as n (%). Continuous variables were compared between two groups via the unpaired Student’s t test (two-tailed). If the Levene test indicated heterogeneity of variance, the Welch test was used. For non-normally distributed parameters, non-parametric Mann-Whitney-U test was used to compare the two groups. Any use of the Welch or Mann-Whitney-U test instead of the t test is explicitly stated in the tables.

**Fig. 5:**
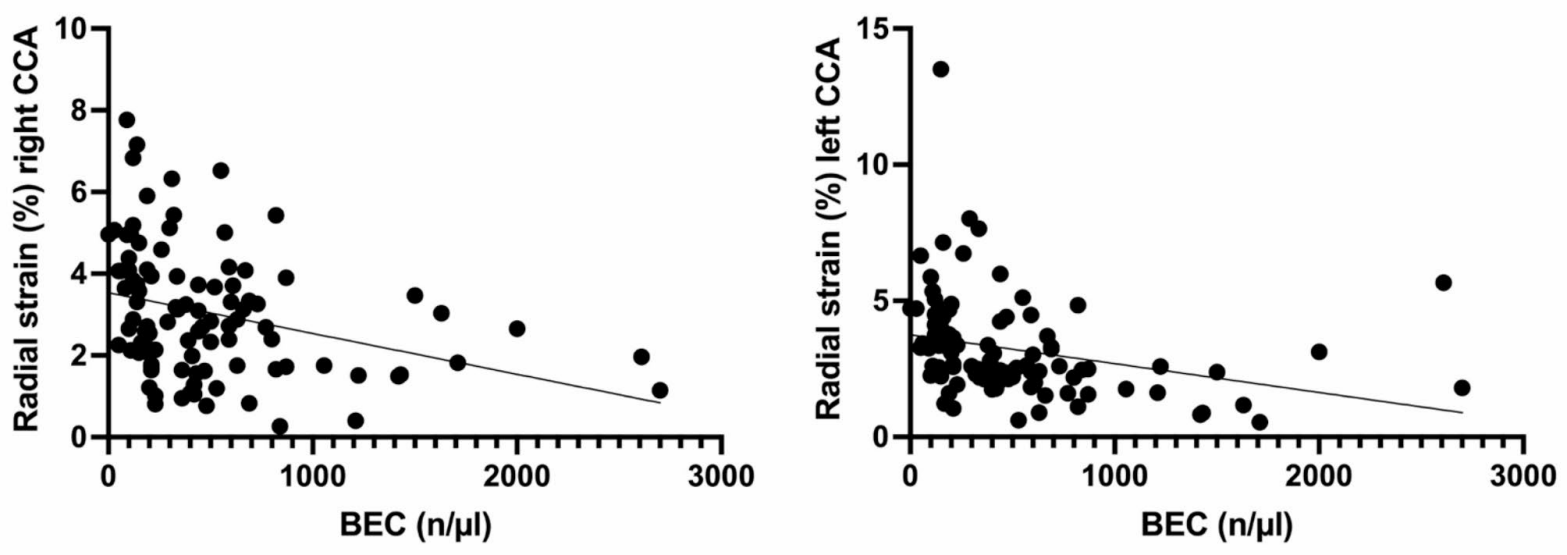
Linear regression analysis adjusted for sex category revealed a small but significant negative correlation between the blood eosinophil count (BEC) and the radial strain values for the right common carotid artery (CCA) (R2 = 0.131, b=-0.001, *p* = 0.001) and left CCA (R2 = 0.086, b=-0.001, *p* = 0.015)

Categorial variables were compared between the two groups via either the chi-square test or Fisher’s exact test, as appropriate.

Additionally, a linear regression analysis was conducted to evaluate the linear correlation between two metric variables. The linear regression was adjusted for sex category. A p value less than 0.05 was considered statistically significant throughout the analysis.

## Results

### Patient population

We enrolled 100 patients in the study, with 58 (58.0%) having a blood eosinophil count (BEC) of ≥ 300/µl. There were no significant differences in age or cardiovascular risk factors; however, patients with BECs < 300/µl were more frequently female (76.2% vs. 51.7%), as indicated in Table 1. The patient group with BECs ≥ 300/µl presented signs of more pronounced disease, as evidenced by lower asthma control test (ACT) scores (15.9 ± 5.5 vs. 13.3 ± 5.9, *p* = 0.038), a higher rate of annual exacerbations (1.3 ± 2.1 vs. 2.6 ± 2.4 n/year, *p* = 0.005), a lower forced expiratory volume in one second (FEV1% predicted, 77.7 ± 19.6 vs. 65.5 ± 18.9, *p* = 0.002) and a higher rate of biologic therapy (2.4% vs. 50.0%, *p* < 0.001). Other asthma medication was similar in both groups.

Baseline characteristics are detailed in Table 1.

### Vascular assessments

Carotid artery ultrasound revealed a similar rate of plaque formation in patients with high or low eosinophil counts (BEC ≥ 300 n/µl: *n* = 29, 50% vs. BEC < 300/µl: *n* = 13, 31.0%, *p* = 0.057).

The analysis of vascular strain revealed that patients with a BEC of ≥ 300 n/µl experienced increased arterial stiffness compared with those with a BEC < 300 n/µl. Specifically, the group with BEC ≥ 300 n/µl had significantly reduced radial strain values (mean radial strain of the right CCA: 2.7 ± 1.4% for BEC ≥ 300 n/µl vs. 3.5 ± 1.7% for BEC < 300 n/µl, *p* = 0.008; mean radial strain of the left CCA: 2.6 ± 1.4% vs. 4.1 ± 2.2%, *p* < 0.001), as illustrated in Fig. 2. Similar results were found for the radial strain rate. The mean radial strain rate of the right CCA was 0.23 ± 0.09 1/s (BEC < 300 n/µl), whereas it was 0.28 ± 0.10 1/s (BEC ≥ 300 n/µl), *p* = 0.006. The left CCA had a mean radial strain rate of 0.23 ± 0.13 1/s vs. 0.32 ± 0.15 1/s (*p* = 0.006), as illustrated in Fig. 3. The rotational strains and rotational strain rates did not significantly differ (rotational strain right CCA: 2.7 ± 1.6% vs. 3.5 ± 2.3%, *p* = 0.053, Welch test). Left CCA: 3.2 ± 1.9% vs. 3.9 ± 2.1%, *p* = 0.099. Rotational strain rates: right CCA: 0.18 ± 0.10 1/s vs. 0.23 ± 0.14 1/s, *p* = 0.085, Welch test. Left CCA: 0.21 ± 0.13 1/s vs. 0.24 ± 0.11 1/s, *p* = 0.186), as depicted in Fig. 4. There was also no significant difference in the vessel wall velocity (right CCA: 0.07 ± 0.04 cm/s vs. 0.08 ± 0.05 cm/s, *p* = 0.330. Left CCA: 0.08 ± 0.04 cm/s vs. 0.08 ± 0.04 cm/s, *p* = 0.850) and displacement (right CCA: 0.10 ± 0.06 mm vs. 0.12 ± 0.07 mm, *p* = 0.111. Left CCA: 0.11 ± 0.06 mm vs. 0.12 ± 0.06 mm, *p* = 0.469), as shown in Fig. 4.

### Linear regression analysis

Linear regression analysis, after adjusting for sex category, indicated a modest negative correlation between the eosinophil cell count and the radial strain for both the right (R2 = 0.131, b=-0.001, *p* = 0.001) and left CCAs (R2 = 0.086, b=-0.001, *p* = 0.015), as illustrated in Fig. 5.

## Discussion

Our findings reveal, for the first time, a link between blood eosinophil count in asthma patients and increased vascular stiffness, suggesting an early indication of atherosclerosis, even in the absence of apparent cerebrovascular disease.

Asthma has been identified as a contributing factor to atherosclerotic conditions, including stroke and heart disease [3, 6]. This linkage is attributed primarily to systemic inflammation, as several other systemic inflammatory conditions are similarly associated with atherosclerosis [12–15]. Atherogenesis is widely considered a type 1 inflammatory process [16, 17]. However, recent attention has shifted toward type 2 inflammation in the context of atherogenesis, driven by clinical observations linking allergic conditions to cardiovascular diseases [18]. Nonetheless, there is a paucity of data regarding atherosclerosis, especially in the context of eosinophilic asthma.

Our study aligns with prior research demonstrating arterial vascular changes in individuals with asthma, including increased intima‒media thickness of the carotid artery and/or increased vascular stiffness of the carotid artery [19–21]. The increased vascular stiffness, which can be measured as reduced strain values, does not necessarily indicate manifest atherosclerosis but should primarily be considered as pre-atherosclerotic changes, which could eventually develop into atherosclerosis over time. This likely explains why the rate of manifest atherosclerosis in our studied population did not differ.

Additionally, cerebrovascular disease has been associated with asthma [6]. However, the analyses did not include a detailed examination of blood eosinophil counts. In our patient cohort, no disparity in the incidence of established cerebrovascular disease was observed between the two groups.

Eosinophil granulocytes are potential mediators linking bronchial asthma and atherosclerosis because of their involvement in type 2 inflammation. Clinical investigations have revealed a correlation between eosinophils and eosinophil cationic protein, an effector protein of eosinophils, and the severity of coronary heart disease [22–24]. Additionally, studies have demonstrated a link between eosinophil levels and mortality rates following coronary intervention [25]. Moreover, a low eosinophil count has been inversely associated with peripheral arterial disease (PAD) [26]. This association has been observed not only in clinical studies but also in a mouse model, where eosinophils were found to exert a pro-atherogenic effect [9].

To our knowledge, few studies have directly evaluated the impact of eosinophil asthma on cardiovascular disease (CVD), but studies have associated adult-onset asthma with CVD and an increased risk of CVD events [27, 28]. Adult-onset asthma frequently correlates with eosinophilia and other markers indicative of type 2 inflammation [10]. Notably, these studies did not provide information on the blood eosinophil count.

Nevertheless, conflicting findings have emerged. For example, an observational study reported a negative association between eosinophils and coronary heart disease (CHD), and higher levels of IL-5 were linked to reduced subclinical atherosclerosis [29, 30].

Mouse models have revealed a potential link between allergic lung inflammation and the development of abdominal aortic aneurysms (AAAs), which represent an advanced stage of atherosclerosis. Interestingly, eosinophils were observed to exert a protective effect against AAA formation in this model, whereas anti-IgE therapy was effective in suppressing the development of AAA [31, 32]. These findings prompt further investigation into the relationships among allergic asthma, the IgE inflammatory pathway, and the mechanisms underlying atherogenesis in asthma.

While our study provides valuable insights, it is subject to certain limitations that warrant consideration. First, its single-center design may introduce bias toward patients with more severe illness, given that patients were recruited from the outpatient department of a university hospital. However, this potential bias would likely affect both groups equally. Additionally, the observed differences in disease severity between individuals with elevated eosinophil counts and those without may have contributed to the results as well. However, eosinophilic asthma usually presents with increased disease severity and more frequent exacerbations. These differences and the natural fluctuations in eosinophil levels highlight the complexity of patient stratification and align with the expected patient group. To address this issue further, further studies with matched controls would be helpful.

It is important to acknowledge the absence of universally accepted threshold values for defining eosinophilic asthma, as well as the inherent variability in blood eosinophil levels intra- and inter-individually. Our patient stratification followed current guidelines to the best of our ability.

While vessel strain analysis does not directly identify established atherosclerosis, it serves as a reliable method for detecting early atherosclerotic changes, surpassing conventional measures of vascular stiffness [33–35].

In conclusion, our findings provide novel insights into the association between eosinophilic asthma and increased vascular stiffness, indicating the potential involvement of eosinophils in atherosclerosis. However, the underlying mechanisms driving this correlation are multifaceted and warrant further investigation for a more comprehensive understanding.

## Data Availability

Data is available from the corresponding author upon reasonable request.

## Abbreviations

AAA: abdominal aortic aneurysm
ACT: asthma control test
BMI: body mass index
BEC: blood eosinophil count
CCA: common carotid artery
CHD: coronary heart disease
CV: cardiovascular
CVD: cardiovascular disease
ECG: electrocardiogram
EGPA: eosinophilic granulomatosis with polyangiitis
FEV1: forced expiratory volume in one second
FeNO: fraction of exhaled nitric oxide
IgE: Immunoglobuline
IL-5-(R): interleukine 5 (receptor)
PAD: peripheral arterial disease
s: second
